# An optimized microfabricated platform for the optical generation and detection of hyperpolarized ^129^Xe

**DOI:** 10.1038/srep43994

**Published:** 2017-03-07

**Authors:** Daniel J. Kennedy, Scott J. Seltzer, Ricardo Jiménez-Martínez, Hattie L. Ring, Nicolas S. Malecek, Svenja Knappe, Elizabeth A. Donley, John Kitching, Vikram S. Bajaj, Alexander Pines

**Affiliations:** 1 Materials Science Division, Lawrence Berkeley National Laboratory, Berkeley, California, USA; 2 Department of Chemistry, University of California at Berkeley, Berkeley, California, USA; 3 Time and Frequency Division, National Institute of Standards and Technology, Boulder, Colorado, USA; 4 Department of Physics, University of Colorado at Boulder, Boulder, Colorado, USA

## Abstract

Low thermal-equilibrium nuclear spin polarizations and the need for sophisticated instrumentation render conventional nuclear magnetic resonance (NMR) spectroscopy and imaging (MRI) incompatible with small-scale microfluidic devices. Hyperpolarized ^129^Xe gas has found use in the study of many materials but has required very large and expensive instrumentation. Recently a microfabricated device with modest instrumentation demonstrated all-optical hyperpolarization and detection of ^129^Xe gas. This device was limited by ^129^Xe polarizations less than 1%, ^129^Xe NMR signals smaller than 20 nT, and transport of hyperpolarized ^129^Xe over millimeter lengths. Higher polarizations, versatile detection schemes, and flow of ^129^Xe over larger distances are desirable for wider applications. Here we demonstrate an ultra-sensitive microfabricated platform that achieves ^129^Xe polarizations reaching 7%, NMR signals exceeding 1 μT, lifetimes up to 6 s, and simultaneous two-mode detection, consisting of a high-sensitivity *in situ* channel with signal-to-noise of 10^5^ and a lower-sensitivity *ex situ* detection channel which may be useful in a wider variety of conditions. ^129^Xe is hyperpolarized and detected in locations more than 1 cm apart. Our versatile device is an optimal platform for microfluidic magnetic resonance in particular, but equally attractive for wider nuclear spin applications benefitting from ultra-sensitive detection, long coherences, and simple instrumentation.

Nuclear magnetic resonance (NMR) spectroscopy and magnetic resonance imaging (MRI) are workhorse techniques in a wide variety of applications, owing to nuclear spins’ long coherence times and extreme sensitivity to their biological, chemical, and physical environments. Unfortunately, conventional NMR and MRI yield small signals arising from room temperature thermal-equilibrium nuclear spin polarizations which never exceed ~10^−5^ even at the very high magnetic fields afforded by superconducting magnets. Moreover, conventional inductive detection requires high magnetic fields^1^ and creates a further reliance on superconducting magnets. Thus, conventional magnetic resonance techniques are typically relegated to sophisticated research environments or dedicated clinical centers.

Signal-to-noise may be enhanced using non-equilibrium “hyperpolarization” methods and/or highly sensitive non-inductive magnetic field detectors. Dynamic nuclear polarization (DNP)^2^, parahydrogen-induced polarization (PHIP)^3^,^4^, and spin-exchange optical pumping (SEOP)^5^ generate long-lived non-equilibrium nuclear spin polarizations and NMR signals up to 10^5^ times larger than equilibrium techniques. Quantum magnetic field sensors such as superconducting quantum interference devices (SQUIDs)^6^ and alkali vapor atomic magnetometers (AVAMs)^7^,^8^ remain highly sensitive even at microtesla magnetic fields.

^129^Xe gas can be hyperpolarized to levels approaching unity^9^,^10^,^11^ and has found use in chemical and biological applications^12^ such as perfusion imaging of the lung^13^, molecular sensing^14^ of sub-picomolar quantities of chemical substances^15^,^16^, and the study of ordered materials such as liquid crystals^17^. Coupling ^129^Xe with AVAMs is particularly attractive because similar instrumentation is used for both polarization and detection and AVAMs are inherently more sensitive than inductive detectors at the low-to-moderate fields required for efficient ^129^Xe hyperpolarization, especially for very small device sizes^18^. This also allows both *in situ* and *ex situ* detection modalities for a wide range of applications and operating conditions while using simple optical technologies. *In situ* detection^19^,^20^, in which the magnetic field sensor is collocated with the ^129^Xe gas, is three orders of magnitude more sensitive due to the 500-fold enhanced magnetization provided by the Fermi-contact hyperfine interaction^21^ and the avoidance of losses due to separation between the magnetic field source and detector. Meanwhile, *ex situ* detection enables detection under a much wider variety of conditions^7^.

We recently developed a proof-of-concept microdevice capable of producing hyperpolarized ^129^Xe gas^22^ for easy integration into microfluidic devices^23^. This device was limited by 0.5% ^129^Xe polarizations and 1 s ^129^Xe relaxation time, operated only using *in situ* detection, and showed degraded performance after a few days. Here we demonstrate a device achieving polarizations of 7% with spin coherence times longer than 6 s while leveraging simultaneous *in situ* and *ex situ* detection. Using *in situ* detection, we demonstrate NMR single-shot detection with signal-to-noise of 10^5^. We study a wide range of operating conditions and identify the optimum conditions for ^129^Xe polarization and NMR signal. We maintain significant polarization during transport over 1 cm, a length scale commensurate with most microfluidic devices. We continuously operate the device for weeks without significant degradation in performance. We find that *in situ* detection provides a signal enhancement by a factor between 2000 and 5000, depending on device geometry. These results demonstrate that ^129^Xe can be efficiently polarized and detected in a wide range of conditions using an integrated single-chip platform. When combined with methodological developments in microfluidic remote detection NMR^24^, these results should greatly improve the prospects for the use of fully integrated ^129^Xe NMR instrumentation in small, portable, inexpensive microfluidic devices.

## Results

### Microfabricated Xe platform

**Figure 1:**
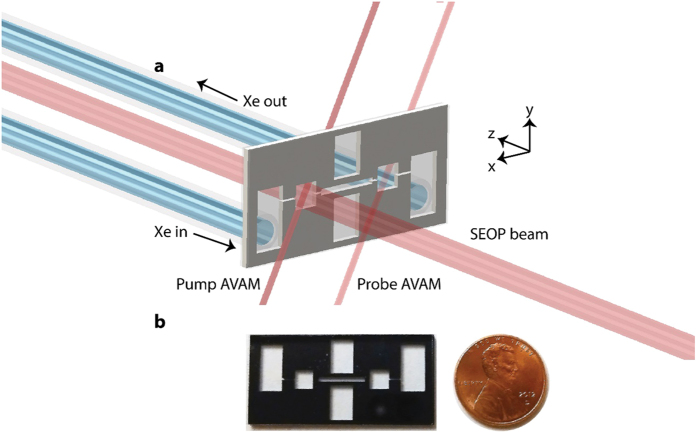
Microfabricated ^129^Xe polarizer. (**a**) A mixture of Xe and N_2_ gas flows from an external gas manifold into the chip. Circularly polarized 795 nm laser light optically pumps a hot Rb vapor in the pump chamber. As ^129^Xe flows through the pump chamber, it is polarized by spin exchange interactions with the optically pumped Rb. Additional 795 nm laser beams are used to optically probe the magnetic field in the pump and probe chambers, while causing minimal perturbation to the polarization state; the beams enter the chambers at 45° from normal to the chip surface. (**b**) The silicon chip has lateral dimensions of 39 mm × 19 mm, with a thickness of 1 mm. The pump and probe chambers both have lateral dimensions of 4 mm × 4 mm. The microchannel connecting the pump and probe chambers is 10 mm long × 1 mm wide. Two tall grooves are etched from the middle of the chip to thermally isolate the two sides of the device. The outside chambers act as attachment sites for glass connections to an external vacuum and gas handling system and have lateral dimensions of 5 mm × 10 mm.

Figure 1 shows one of the devices used in this work. Briefly, the devices are fabricated by etching holes and grooves from a silicon wafer and bonding glass windows to both sides of the silicon, forming a series of chambers and channels. ^129^Xe flows into the device and is polarized through spin-exchange optical pumping (SEOP)^5^ with Rb vapor polarized using a distributed feedback (DFB) laser. The polarized ^129^Xe flows through a 1 cm microfluidic channel, experiences magnetic field pulses, and is optically detected using Rb vapor and another DFB laser configured to operate as a zero-field magnetometer (see Methods). In comparison to our previous work^22^ the device presented here incorporates novel fabrication methods, which we believe have enabled the versatile operation and highly improved performance described below. Previously, Rb was prepared by a reaction between rubidium chloride and barium azide under vacuum; this procedure produces a barium chloride film as a byproduct^25^. We believe that this contributed to accelerated nuclear spin relaxation in our previous device. In this study, Rb was prepared by photolysis of rubidium azide, which produces no lingering chemical byproducts^26^,^27^. Additionally, attachment of the device to the vacuum and gas handling system was accomplished by glassblowing techniques instead of high vacuum epoxies. A second design incorporating a sealed second probe chamber was utilized in some experiments. This design was utilized only when specifically referred to in the discussion that follows. A detailed description of the device design and general experimental procedures are provided in the Methods section.

### ^129^Xe NMR Signal, Polarization, and Relaxation Time

**Figure 2:**
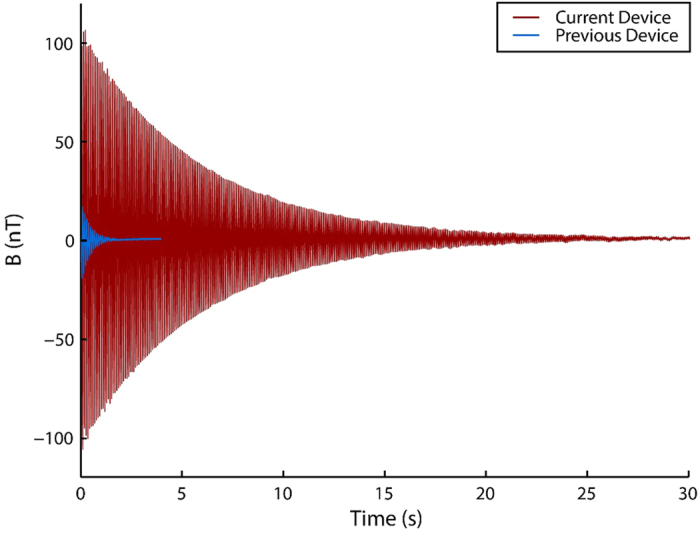
^129^Xe NMR signals from the first-generation (blue) and second-generation (red) devices at similar conditions. Both devices operated at approximately 140 °C, used a 200 Torr partial pressure of Xe gas at natural isotopic abundance (26.4% ^129^Xe), a N_2_ partial pressure of 400 Torr, and 8 mW of optical pumping power. In the first-generation device, optical pumping was applied for 10 s prior to measurement. For the second-generation device, the optical pumping duration was 30 s. The blue curve is reproduced from our previous work^22^.

The fabrication procedures described above lead to substantial improvements in device performance. Figure 2 shows the ^129^Xe NMR signals obtained in the pump chambers using the original and new fabrication techniques with similar gas mixtures (400 Torr N_2_, 200 Torr Xe at natural isotopic abundance, 1 Torr = 133.3 Pa), temperatures (140 °C), and optical powers (8 mW), under stopped flow conditions. These fabrication improvements allow for the generation of a contact-interaction-enhanced NMR signal with amplitude of 110 nT, polarization of 3.5%, and T_2_ relaxation time of 6.15 s, in comparison to 20 nT, 0.7%, and 1.25 s, respectively, obtained in the previous device^22^, when the two devices are operated under similar conditions. These results represent a significant improvement in device performance and are comparable with those achieved in the best sealed microfabricated devices^28^, which are incapable of delivering hyperpolarized ^129^Xe to another location.

### Simultaneous *In Situ* and *Ex Situ* Magnetometry

The use of AVAMs in NMR has typically employed *ex situ* measurement schemes, in which a sealed AVAM is located at a position physically proximate to, but still distinct from, the system being measured^7^. The monolithic nature of our device also allows for operation using an *in situ* detection modality in which the alkali atoms used to measure the NMR signal are in the same physical location as the hyperpolarized ^129^Xe generating the signal. This *in situ* scheme results in a significant enhancement of the resultant signal due to a combination of the 500-fold signal increase afforded by the Fermi-contact interaction^21^ and the avoidance of dipolar losses associated with the use of a detector located some distance away from the magnetic field source. While the *in situ* detection modality provides very large signal-to-noise, it cannot be used in situations where the ^129^Xe gas is dissolved into an aqueous or organic solvent, resulting from chemical oxidation of the magnetometer alkali atoms. Because of this, we view the *in situ* and *ex situ* detection modalities as complementary techniques, and sought to characterize the performance of both methods.

**Figure 3:**
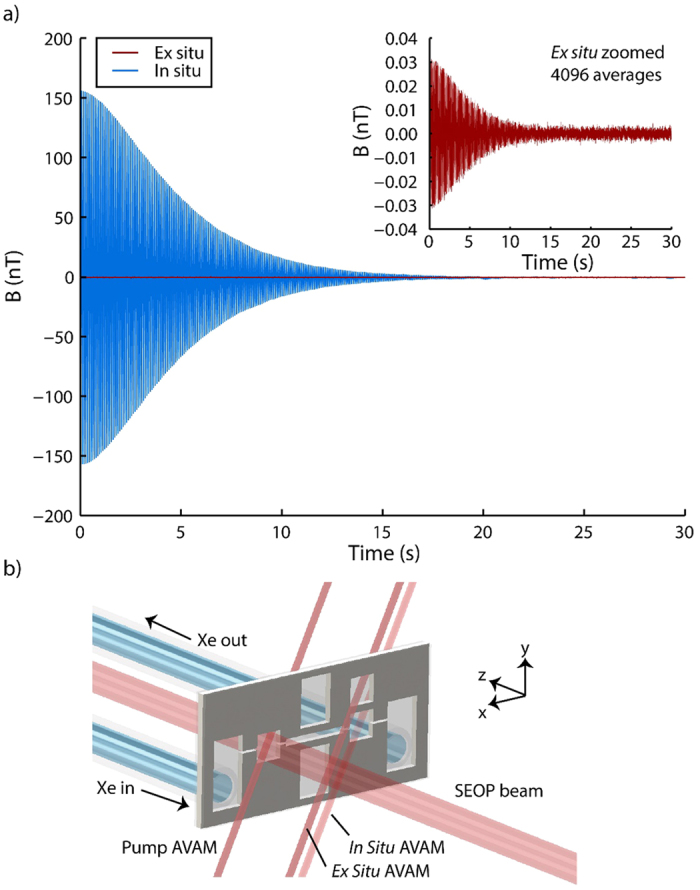
Measurement of *in situ* magnetometer signal enhancement. (**a**) ^129^Xe NMR signals in the *in situ* (blue) and *ex situ* (red) magnetometer chambers. Inset: Zoom-in of the *ex situ* magnetometer signal, averaged 4096 times. In all cases, the pump chamber was maintained at a temperature of 140 °C and an optical pumping power of 70 mW was employed. A gas mixture consisting of 800 Torr Xe at natural isotopic abundance (26.4% ^129^Xe) and 800 Torr N_2_ was flowed through the device at a rate of 5 μL s^−1^. The magnetometer chambers were maintained at a temperature of 110 °C and an optical power of 100 μW was employed in each magnetometer chamber. (**b**) Geometry of device combining *in situ* and *ex situ* magnetometry modalities. The *ex situ* magnetometer has dimensions identical to those of the *in situ* chamber and is separated from the gas flow by 1 mm of silicon in the *y* direction.

We performed simultaneous *in situ* magnetometry using the integrated probe chamber, and *ex situ* magnetometry in an identical but sealed chamber etched a distance of 1 mm away from the probe chamber. The probe DFB laser was tightly focused, optically isolated, linearly polarized, and directed to a beam displacer crystal. The beam displacer produced parallel beams with perpendicular linear polarizations separated by a distance of 5 mm, allowing each beam to be directed through the same quarter-wave plate and into the center of the proper magnetometer cell. Using this setup, the two magnetometers displayed sensitivities which were equal to within 2%; each chamber attained a magnetic field sensitivity of approximately 4 pT Hz^−1/2^ in a 40 Hz bandwidth. The main part of Fig. 3 shows the single-shot NMR signals attained in the two magnetometers. Due to the low signal-to-noise ratio in the *ex situ* magnetometer, we averaged the *ex situ* signal 4096 times in order to obtain a good estimate of the magnetic field in this chamber; this averaged data is shown in the inset of Fig. 3. The *in situ* magnetometer provides a signal enhancement by a factor of 5300 ± 200 over that obtained in the *ex situ* magnetometer. This number is in agreement with numerical simulations conducted using the COMSOL Multiphysics 5.0, Magnetic Fields module (COMSOL Group, Stockholm, Sweden). These simulations also indicate that the *in situ* magnetometer should provide a signal enhancement by a factor of approximately 2400 even when an *ex situ* magnetometer in closer proximity is used. A description of these simulations is provided in Supplementary Note 1.

### Device optimization

**Figure 4:**
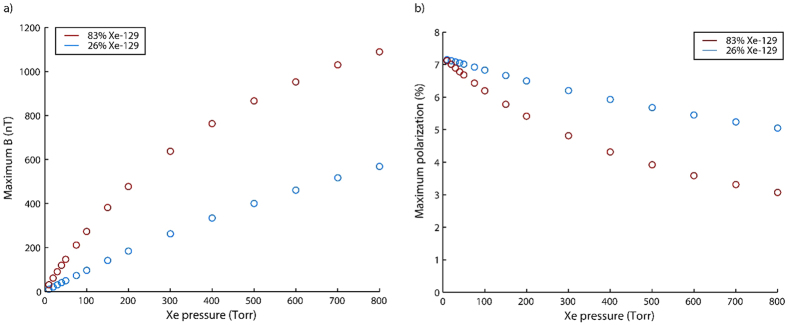
(**a**) Maximum attained ^129^Xe magnetic field for various Xe partial pressures. (**b**) Maximum attained ^129^Xe polarization for various Xe partial pressures. In both (**a**) and (**b**), data in blue represent Xe gas at natural isotopic abundance (26.4% ^129^Xe) while data in red represent isotopically enriched Xe gas containing 83% ^129^Xe. Within the error bars, the maximum polarizations and fields were generally obtained at the highest optical pumping power of 70 mW. Data were obtained in the pump chamber under stopped-flow conditions. In all cases, a N_2_ partial pressure of 800 Torr was employed. All experiments were conducted at a temperature of approximately 140 °C. A set of data consisting of the fields and polarizations obtained as a function of optical pumping power for each Xe partial pressure is presented in Supplementary Figure 2.

While some applications rely on high polarization levels, others benefit from large ^129^Xe magnetization, which can be achieved by increasing ^129^Xe density while sacrificing polarization. To characterize these two regimes, we have studied the conditions which optimize the ^129^Xe polarization and NMR signal in the pump chamber while under stopped-flow conditions. Supplementary Figure 2 shows the NMR signals and polarizations for a variety of gas compositions at different optical pumping powers. As discussed below, the device operates over a significantly longer lifetime when the gas mixture is maintained above atmospheric pressure. To this end, all of the characterization experiments utilized a N_2_ partial pressure of 800 Torr. Figure 4 presents these data as the maximum attainable NMR signals and polarizations at each Xe partial pressure. 10 Torr of Xe at natural isotopic abundance (26.4% ^129^Xe) allows the highest polarizations of up to 7% but yields an NMR signal of only 10 nT. In contrast, the use of 800 Torr isotopically enriched Xe (83% ^129^Xe) yields a polarization of just less than 3% and an NMR signal of more than 1000 nT. Supplementary Figure 3 shows the NMR signals in the pump and probe chambers as a function of total gas flow rate. It should be noted that the highest polarizations of 7% greatly exceed those obtainable in the first-generation device^22^, which had a polarization limited to less than 1% due to shorter ^129^Xe lifetimes.

### Enhanced Device Lifetime at High Pressures

**Figure 5:**
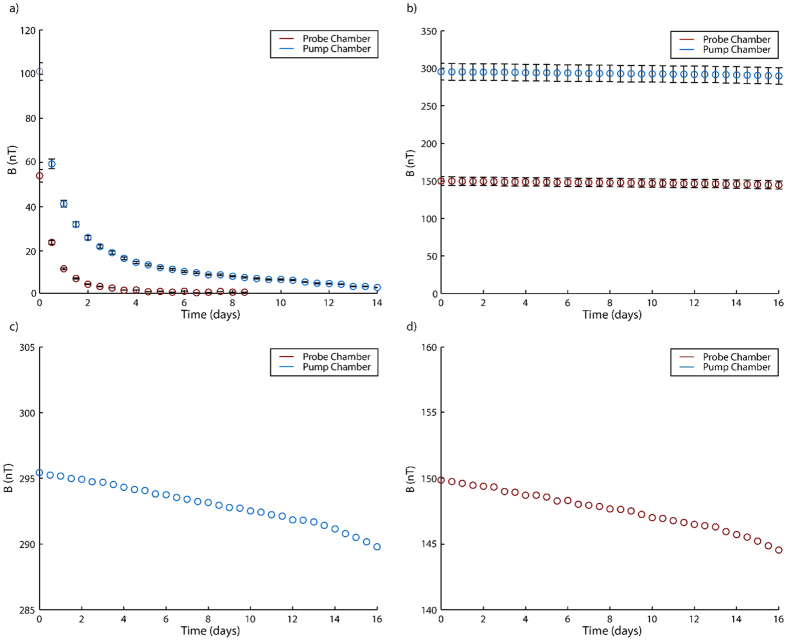
Decay of ^129^Xe signal during continuous device operation. (**a**) Device operated continuously under conditions similar to those employed in first-generation device: 200 Torr Xe at natural isotopic abundance (26.4% ^129^Xe), 400 Torr N_2_. (**b**) Device operated continuously under 800 Torr Xe at natural isotopic abundance, 800 Torr N_2_. (**c**) Zoom-in on pump chamber signal (same data as in **b**). (**d**) Zoom-in on probe chamber signal (same data as in **b**). In all cases, the devices were continuously operated under a total gas flow rate of 5 μL/s and a temperature of approximately 140 °C. Pump chamber signals are shown in blue and probe chamber signals are shown in red.

Operation at sub-atmospheric pressures leads to very short device lifetimes; over the course of a few days of operation, the NMR signal intensity and lifetime decreased rapidly until signals became unobservable. We hypothesize that this is due to the slow leak of gases (mainly oxygen and water vapor) into the device; over time, these gases oxidize Rb and form adsorptive films on the chamber walls, causing enhanced wall relaxation. Assuming that O_2_ is the major oxidative component, that Rb_2_O is the end product, and that the resulting film has a structure similar to the crystal structure of Rb_2_O, we calculate that an ultrahigh-vaccum (UHV) standard leak rate of 10^−9^ Torr L s^−1^ produces a monolayer on the glass walls of the pump and probe chambers approximately every 10 h. To avoid this formation of Rb_2_O, a process we refer to as “aging” of the device, we operated the device at pressures greater than 1 atm to ensure that all leaks would flow out of the device rather than into it. Figure 5a shows the signal of a device operated continuously at a total gas pressure of 600 Torr (200 Torr Xe at natural abundance, 400 Torr N_2_) and a flow rate of 5 μL s^−1^, while Fig. 5b shows a device operated at a total gas pressure of 1600 Torr (800 Torr Xe at natural abundance, 800 Torr N_2_) at the same flow rate. Whereas the device ages rapidly when operated at 600 Torr, the device operated at 1600 Torr shows a slow degradation in the maximum attainable ^129^Xe signal over the course of 16 days of continuous operation, retaining more than 96% of the signal amplitude at the point at which the device is first activated. Operation at 1600 Torr ended when one of the glass tubes connecting the device to the flow manifold was broken by operator error, exposing the system to the atmosphere.

## Discussion

Although we have achieved the highest reported ^129^Xe polarization in a microfabricated device, large-scale polarizers may obtain polarizations an order of magnitude higher^11^. This difference is due to the relatively short ^129^Xe relaxation times which result from increased wall collisions in a device with a very high surface-area-to-volume ratio. In contrast, large-scale polarizers tend to use pumping cells with large volumes and very long relaxation times, allowing for high polarizations to be achieved. In a “batch mode” model of production, in which hyperpolarized ^129^Xe is produced and collected for later use, high ^129^Xe polarizations are generally desirable. However, this “batch mode” production model is time-consuming, requiring long periods for the buildup of ^129^Xe polarization (on the order of the spin relaxation time), collection of large amounts of gas, and transport to the site of use. In comparison, the “continuous flow” model implemented in our device uses less highly polarized ^129^Xe as it is produced. In such systems, it may be beneficial to sacrifice nominal ^129^Xe polarization in order to produce large quantities of less highly polarized gas and optimize the NMR signal instead of the polarization. For instance, Fig. 2 shows that operation of our device at a high Xe partial pressure produces NMR signals more than 100 times larger than those obtained using low Xe partial pressures while only reducing ^129^Xe polarization by a factor of approximately 2. To this end, the relatively short relaxation lifetimes and attendant low polarizations in our device may actually be beneficial to certain applications, in that they would allow for more rapid or sensitive NMR experiments. At the very least, these considerations suggest that ^129^Xe polarization is an incomplete measure of polarizer performance. To better compare our device with large-scale polarizers, we look at two figures of merit for a ^129^Xe polarizer: the volume rate of production of hyperpolarized ^129^Xe and the spin-transfer efficiency^29^. The latter is a measurement of the efficiency with which angular momentum is transferred from photons to ^129^Xe and is likely to be an especially critical figure in future low-power, fully microfabricated polarizers. Interestingly, we find that the volume rate of production increases with increasing flow rate, even as the polarization decreases. At a flow rate of 5 μL s^−1^ and using a gas mixture containing 800 Torr natural abundance Xe, we achieve a polarized ^129^Xe production rate of 0.97 μL s^−1^ W^−1^. Using the same gas mixture under stopped-flow conditions, we achieve a spin-transfer efficiency of 0.005. These results compare favorably with, for instance, figures of 30% ^129^Xe polarization, 0.45 μL s^−1^ W^−1 129^Xe production rate, and 0.005 spin-transfer efficiency obtained in a recent large-scale polarizer^11^. It is important to note that a volume production rate one order of magnitude higher has been reported using a large-scale polarizer with a helical xenon flow path^30^.

Numerous recent developments suggest the possibility of even longer relaxation times, polarizations, and NMR signals, as well as future applications. The recent development of low-temperature bonding techniques^31^,^32^ has allowed the use of antirelaxation coatings^33^,^34^ in microfabricated vapor cells for atomic clocks and magnetometers^35^ and may allow for longer relaxation times and polarizations in future microfabricated ^129^Xe polarizers. Minimization of supporting systems such as gas handling devices and optical components might benefit from ongoing developments in chip-scale vacuum pumps^36^,^37^ and microfluidic gas handling valves^38^. Also, high-power vertical cavity surface emitting lasers (VCSELs)^39^ might be integrated to significantly reduce the need for large optical elements, similar to their use in microfabricated AVAMs^40^. Vacuum packaging, already developed for AVAMs^41^,^42^, should significantly reduce convective losses and power requirements for a future fully portable device.

Of particular importance is the development of methods allowing the *in situ* detection scheme to be implemented in a greater number of applications. While the *in situ* configuration provides a very large signal enhancement, it places strict restrictions on the nature of the sample being evaluated. Due to the extreme chemical reactivity of alkali atoms and the very small sample sizes, it is important that any ^129^Xe sample being detected through an *in situ* measurement be completely free of any reactive chemical species. This currently limits the *in situ* detection modality to use in very clean applications, as might be encountered if the device is used for remotely detected gas-phase imaging of solid-state microstructures^43^ or the preparation of nuclear targets^44^,^45^. For other applications, it is currently necessary to operate an external magnetometer in the much more versatile *ex situ* detection modality and forgo the large signal enhancement afforded by *in situ* detection. Although we intend for this device to be a generic platform for ^129^Xe NMR measurements, we hypothesize that it will find particularly extensive use in microfluidic ^129^Xe biosensing applications. In such a scenario, hyperpolarized ^129^Xe will generally be dissolved into water or another solvent, magnetically encoded, and read out using a magnetometer. To utilize the *in situ* technique in such a system, it will be necessary to cleanly dissolve ^129^Xe into water and extract it after the application of the relevant NMR experiment. A promising direction for this work is the use of functionalized thin film membranes which might allow for the passage of gases while blocking the flow of solvent molecules; such techniques may allow for the extraction of ^129^Xe from solution^46^,^47^, providing the clean magnetically encoded ^129^Xe required for *in situ* detection. Other approaches to separating the liquid and gas might include the integration of surface-modified silicon nanopillars^48^. We believe that such research might allow far more versatile detection schemes while still leveraging the very large signal enhancements afforded by the *in situ* technique.

## Conclusion

We have presented a microdevice composed of glass and silicon which is capable of optically producing and detecting hyperpolarized ^129^Xe gas for use in magnetic resonance experiments. Using this device, we achieved polarizations up to 7%, fields greater than 1 μT, and T_2_ relaxation times longer than 6 s. We measured a > 5000-fold signal enhancement using a combination of the Fermi-contact interaction and 100% filling factor in an *in situ* alkali vapor atomic magnetometer. We have shown that the device is capable of operating continuously for a period of at least a few weeks before “aging” processes significantly degrade its performance. Finally, we have presented a simple analysis of the factors which will be of importance in the design of future fully microfabricated hyperpolarized ^129^Xe production and detection platforms. We believe that such improvements will ultimately result in a small, inexpensive, and portable device which is generally applicable to a wide variety of ^129^Xe NMR and MRI experiments.

## Methods

### Device Design, Microfabrication, and Preparation

The device is fabricated from silicon and glass using a combination of deep reactive-ion etching (DRIE) and anodic bonding in a manner similar to that developed for miniaturized AVAMs^49^. The overall dimensions of the Si pre-form are 39 mm × 19 mm × 1 mm thick. DRIE is used to etch four holes through the Si, which form the inlet, pump, probe, and outlet chambers of the device. The inlet and outlet chambers have lateral dimensions of 5 mm × 10 mm to allow attachment of ultrahigh vacuum connections. The pump and probe chambers have lateral dimensions of 4 mm × 4 mm. DRIE is also used to etch grooves 300 μm wide and 300 μm deep to form channels connecting the chambers. An area with lateral dimensions of 10 mm × 10 mm is placed in the center of the device, between the pump and probe chambers, to allow the implementation of NMR experiments. The two sides of the chip are separated by tall, narrow holes in the Si (again produced by DRIE), thermally isolating the two sides and allowing independent heating of the pump and probe chambers. A piece of 300 μm thick borosilicate glass is anodically bonded to one side of the Si pre-form at a temperature of 400 °C and a potential difference of 1000 V.

To add rubidium metal to the device, we use a procedure similar to that recently developed for microfabricated AVAMs^26^,^27^ With one piece of glass already attached, a micropipette is used to add 4 μL of RbN_3_ (ChemSavers, Inc., Powhatan, VA) at a concentration of 746 mg mL^−1^ in water to the pump and probe chambers while heating to 90 °C. Using this technique, the salt is rapidly dried into the chambers without spattering onto the unbonded silicon surface. Upon photolysis with intense ultraviolet (UV) light of wavelengths below 250 nm, 2 mg of rubidium metal is formed via the reaction 2 RbN_3_ (s) → 2 Rb (s) + 3 N_2_(g). Since N_2_ gas is the only byproduct, this is a clean reaction which leaves no solid residues. Following addition of the RbN_3_, the device is sealed with a piece of 700 μm thick borosilicate glass to which long pieces of glass tubing and 1/4″ (6.35 mm) stainless steel VCR ultrahigh vacuum connectors have previously been added using glassblowing techniques. The sealing process is accomplished via anodic bonding at a reduced temperature of 300 °C and an increased potential difference of 2000 V to avoid premature thermal breakdown of the RbN_3_, which occurs at a temperature of approximately 315 °C. A schematic of the sealed device is shown in Fig. 1.

In one experiment, an *ex situ* magnetometer chamber is etched into the silicon, as shown in Fig. 3. Since this magnetometer is sealed and inaccessible to gas flow, it is filled before the device is connected to the other experimental systems described below. In preparation for this experiment, the device is placed in a glovebox filled with very dry nitrogen and baked on a hotplate at 250 °C for 72 h to remove all water vapor. Approximately 65 μg of rubidium azide is added to the *ex situ* chamber and dried prior to sealing of the device. This quantity is chosen to produce enough nitrogen gas to obtain a pressure-broadened Rb linewidth equal to that of the *in situ* chamber upon activation of the Rb metal, as described below. The device is sealed via anodic bonding inside of the glovebox.

### Experimental Setup

The various experimental support systems are shown in Supplementary Figure 1. The device is attached to an ultrahigh-vacuum (UHV) system through 1/4″ (6.35 mm) 304 L stainless steel VCR connectors. Briefly, it consists of a gas cylinder each for 99.999% N_2_ and Xe gases (either at the natural isotopic abundance of 26.4% ^129^Xe or isotopically enriched to 83% ^129^Xe, Nova Gas Technologies, Inc., Charleston, SC). The cylinders are separately valved and joined at a t-junction to allow arbitrary gas mixtures. The gas mixture is cleaned using an activated metal getter (SAES Getters, S.p.A., Milan, Italy) which reduces oxygen, water, and other impurities to levels below 1 parts-per-billion (ppb). The gas mixture enters the device through an inlet chamber and leaves through an outlet chamber. The total gas flow rate is controlled using a mass flow controller (Bronkhorst USA, Inc., Bethlehem, PA). In all experiments, the flow rate quoted is the flow rate measured at standard conditions (N_2_ at 760 Torr and 0 °C); actual flow rates at the varying experimental conditions can be obtained by correcting for the partial gas pressures and the different thermal conductivities of N_2_ and Xe. The entire vacuum system can be evacuated to vacuum levels below 10^−7^ Torr using a turbomolecular pump (Pfeiffer Vacuum, Inc., Asslar, Germany). Prior to UV activation of the rubidium metal, the vacuum and gas handling system is baked at 200 °C under vacuum for 24 h; the polarizer chip is heated to 150 °C during this bakeout.

The device is placed inside a two-layer μ-metal magnetic shield (MuShield Co., Manchester, NH) and a series of magnetic field coils (manufactured in-house). A Helmholtz coil produces a homogeneous field in the *z* direction, while a pair of saddle coils produce homogeneous fields in the *x* and *y* directions, allowing the production of fields in any direction. A second set of these coils allows application of magnetic field pulses along any direction for manipulation of the ^129^Xe nuclear spins. Heating of the device is accomplished using homebuilt resistive PCB heaters driven by a 120 kHz AC current; separate heaters are used in the pump and probe chambers to allow independent control of the temperature in each chamber. Temperatures are sensed using a thermistor and regulated by a homebuilt PID controller. The studies described herein generally use a temperature of 140 °C in the pump chamber and 110 °C in the probe chamber.

The Rb atoms are manipulated through interaction with distributed feedback (DFB) lasers (Eagleyard Photonics, GmbH, Berlin, Germany) tuned to the D1 electronic transition at 794.7 nm. For SEOP in the pump chamber, the laser light is focused, directed through an optical isolator, circularly polarized, and expanded to fill the entire lateral area of the chamber. Optical powers ranging from 1 mW–70 mW are employed in the pump chamber. Independently controlled DFB lasers are used to make magnetometry measurements in the pump and probe chambers, with one beam for each chamber. For each magnetometer, the light is tightly focused, optically isolated, circularly polarized, reduced in intensity using a series of neutral density filters, and directed into the chamber at a 45° angle to the normal. The experiments described herein generally operated with magnetometer optical powers of 100 μW. We attained sensitivities of 4 pT Hz^−1/2^ in the probe chamber and 6 pT Hz^−1/2^ in the pump chamber. In one experiment, the probe beam is split into two parallel beams of equal intensity using a beam displacer (Thorlabs, Inc., Newton, MA) prior to the circular polarization step; this configuration is shown in Fig. 3. During signal acquisition, SEOP was turned off by blocking the SEOP beam using an electronically-controlled shutter (Thorlabs, Inc., Newton, MA).

### Optical Detection of Hyperpolarized ^129^Xe

The ^129^Xe NMR signal was detected using a similar methodology as that presented in our previous work^21^. Briefly, the AVAMs utilized in this study are implemented in the zero-field configuration^19^,^50^. The magnetic field is modulated along the *y*-axis using a sinusoidal waveform of 1 μT amplitude and 7 kHz frequency. Transmitted laser intensity is measured with a photodiode and extracted by lock-in detection at the modulation frequency. The bandwidth of the signal is limited to 40 Hz by the lock-in response characteristics. The dispersive magnetometer signal is linear over magnetic fields directed along the *y*-axis for fields smaller than the resonance line-width (HWHM linewidth of 0.8 μT at zero-light levels and up to 3 μT with strong optical pumping). The magnetometer sensitivity is determined by varying the magnetic field along the *y*-axis and fitting the on-resonance slope of the dispersive component of the magnetometer signal. This slope allows calibration of the NMR signal amplitude in magnetic field units.

The magnetization produced by the ^129^Xe hyperpolarization process is directed along the *z*-axis, while the AVAMs are sensitive to magnetic fields along the *y*-axis. Thus, detection of the ^129^Xe NMR signals begins with the application of a DC magnetic field pulse along the *x*-axis to rotate the ^129^Xe magnetization into the transverse *xy*-plane. The ^129^Xe free-induction decay (FID) in each chamber is recorded as the ^129^Xe magnetization precesses. An external magnetic field of magnitude approximately 1 μT is directed along the *z*-axis, resulting in a ^129^Xe precession frequency of approximately 12 Hz.

The NMR signal in the pump chamber can be fit to a single exponentially decaying sinusoid, from which we extract the amplitude, decay time constant, and precession frequency. The FID amplitude in the probe chamber is obtained by measuring the amplitude of the first peak and valley in the probe FID signal. This approximate measurement arises because transverse polarization is transferred from the pump chamber to the probe chambers during measurement of the probe signal. Such a complication yields a non-exponential decay of the probe FID, making curve fitting with a single decaying exponential function an approximation. The ^129^Xe polarization is extracted by using the approximate expression^51^ for the magnetic field *B*_*Xe*_ produced by an ensemble of polarized ^129^Xe atoms:


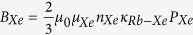


Here, *μ*_0_ is the permeability of free space, *μ*_*Xe*_ is the ^129^Xe nuclear magnetic moment, *n*_*Xe*_ is the ^129^Xe atomic density, *κ*_*Rb*−*Xe*_ is the enhancement factor (of approximately 500)^21^ due to the Fermi-contact hyperfine interaction between the Rb valence electron and the ^129^Xe nucleus, and *P*_*Xe*_ is the ^129^Xe polarization.

## Additional Information

**How to cite this article:** Kennedy, D. J. *et al*. An optimized microfabricated platform for the optical generation and detection of hyperpolarized ^129^Xe. *Sci. Rep.* **7**, 43994; doi: 10.1038/srep43994 (2017).

**Publisher's note:** Springer Nature remains neutral with regard to jurisdictional claims in published maps and institutional affiliations.

## Supplementary Material

Supplementary Information
