# Supplementary material for: An optimized microfabricated platform for the optical generation and detection of hyperpolarized 129Xe
Source: Sci Rep. 2017 Mar 7;7:43994. doi: 10.1038/srep43994 (PMC5339783; doi:10.1038/srep43994)
Supplement: Supplementary Information [file srep43994-s1.doc]

**Supplementary Information for: An optimized microfabricated platform for the optical generation and detection of hyperpolarized ^129^Xe**

Daniel J. Kennedy^1,2,*^, Scott J. Seltzer^1,2,**^, Ricardo Jiménez-Martínez^3,4,***^, Hattie L. Ring^1,2,****^, Nicolas S. Malecek^1^, Svenja Knappe^3^, Elizabeth A. Donley^3^, John Kitching^3^, Vikram S. Bajaj^1,2,*****^, Alexander Pines^1,2^

1) Materials Science Division, Lawrence Berkeley National Laboratory, Berkeley, California, USA

2) Department of Chemistry, University of California at Berkeley, Berkeley, California, USA

3) Time and Frequency Division, National Institute of Standards and Technology, Boulder, Colorado, USA

4) Department of Physics, University of Colorado at Boulder, Boulder, Colorado, USA

5) Department of Physics, University of California at Berkeley, Berkeley, California, USA

* Current Address: Nuclear and Chemical Sciences Division, Lawrence Livermore National Laboratory, Livermore, California, USA

** Current Address: Chevron Energy Technology Company, Houston, Texas, USA

*** Current Address: ICFO-Institut de Ciencies Fotoniques, The Barcelona Institute of Science and Technology, Castelldefels, Barcelona, Spain

**** Current Address: Center for Magnetic Resonance Research, University of Minnesota, Minneapolis, Minnesota, USA

***** Current Address: Google[x], Mountain View, California, USA


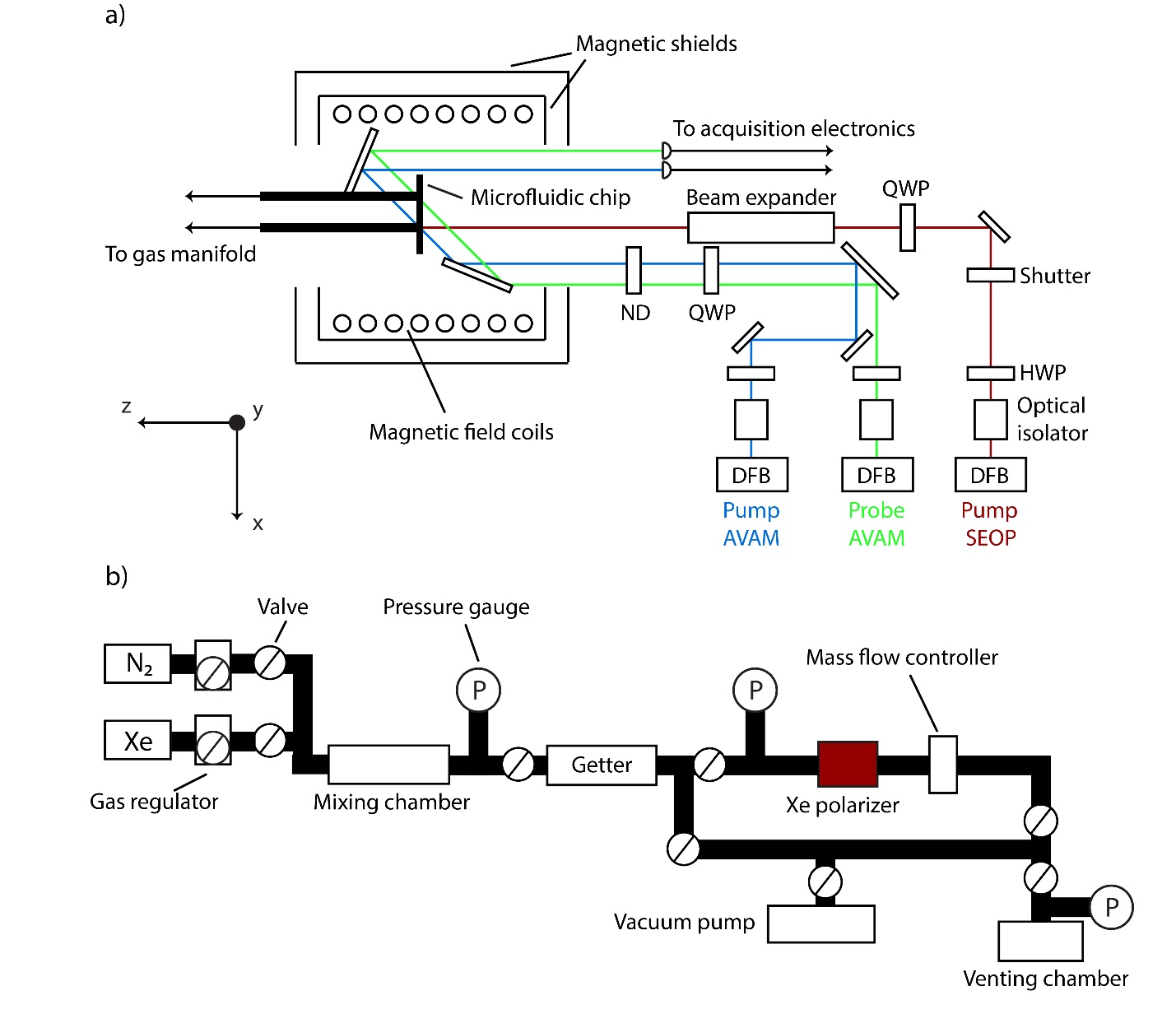


Supplementary Figure 1. ^129^Xe experimental setup. a) Microfluidic chips and optics. Three separate distributed feedback (DFB) lasers produce light at 795 nm for spin-exchange optical pumping (SEOP) in the pump chamber and alkali vapor atomic magnetometry (AVAM) in the pump and probe chambers. The SEOP beam is circularly polarized, expanded to completely fill the pump chamber, and directed into the pump chamber of the microfluidic device. An electronically controlled shutter allows termination of SEOP during magnetometer measurements in the pump chamber. The magnetometer beams are circularly polarized, reduced in intensity using neutral density filters, and directed into the proper chamber at a 45° angle to normal of the chip surface. The optical absorption is detected with a photodiode and the magnetometer signals are extracted by lock-in detection. The microfluidic chip is placed inside of magnetic field coils inside of a two-layer μ-metal magnetic shield. Gas flows into the device through glass tubing connected to an external gas manifold. QWP: quarter-wave plate. HWP: half-wave plate. ND: neutral density filter. b) Gas manifold. Xe and N_2_ are mixed to the desired partial pressures in a mixing chamber. The mixture is cleaned during flow through an activated metal getter. The gas flows through the device at a rate set by a mass flow controller and is collected in a venting chamber which has previously been evacuated. The entire system is evacuated using a turbomolecular pump prior to operation.


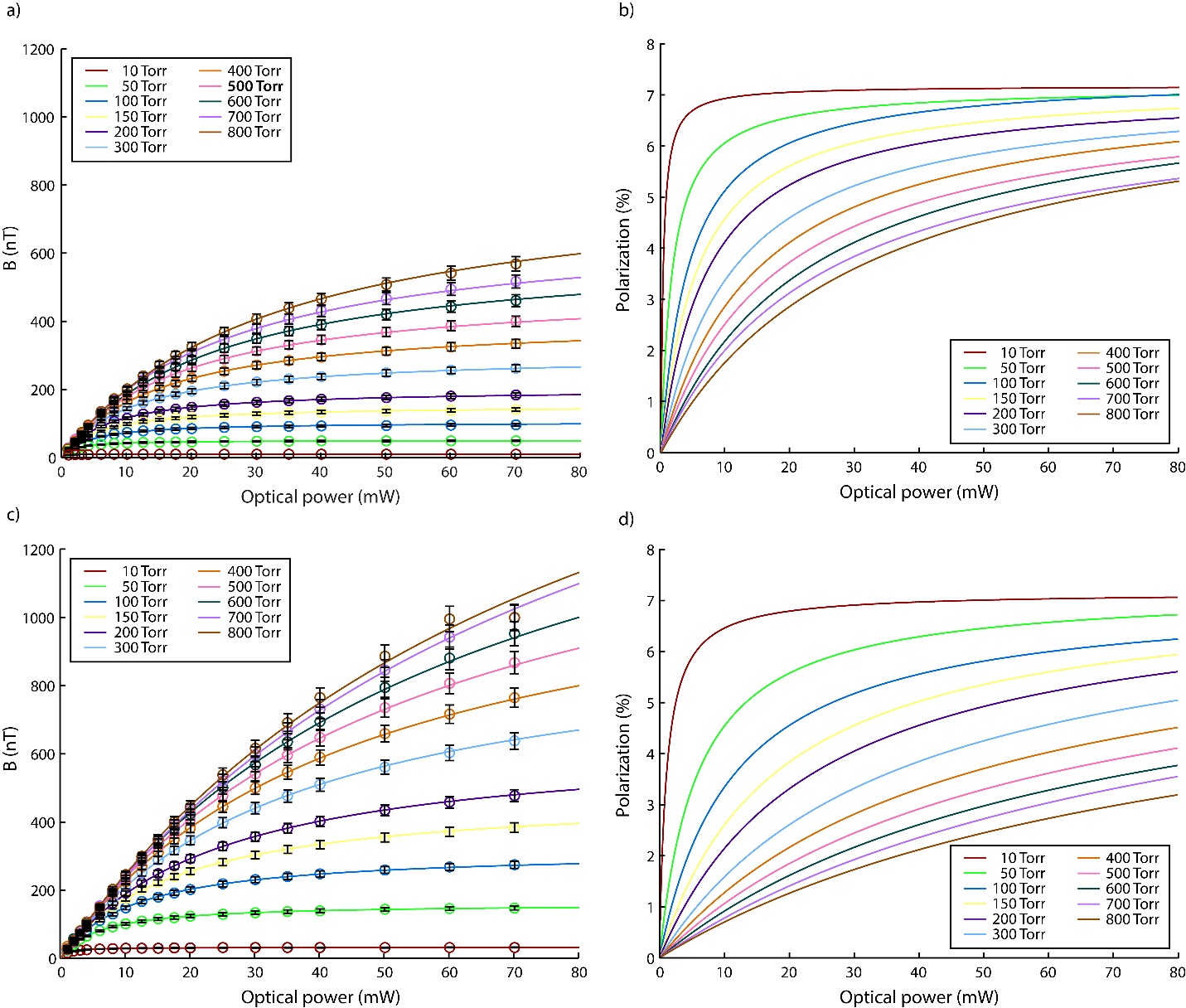


Supplementary Figure 2. ^129^Xe magnetic fields and polarization versus optical pumping power at a variety of Xe partial pressures. In (a) and (b), Xe gas at natural isotopic abundance (26.4% ^129^Xe) was employed. In (c) and (d), isotopically enriched Xe gas containing 83% ^129^Xe was employed. In all cases, a N_2_ partial pressure of 800 Torr was employed. All experiments were conducted at a temperature of approximately 140 °C. Curves in the magnetic field plots were obtained by performing a non-linear least squares fit to a function of the form
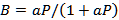
, with
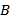
 the magnetic field and
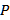
 the optical pumping power^9^. Polarization curves were extracted from these fits using the equation
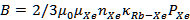
 with
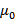
 the permeability of free space,
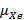
 the ^129^Xe nuclear magnetic moment,
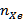
 the ^129^Xe atomic density,
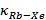
 the enhancement factor (of approximately 500) [Ma 2011] due to the Fermi-contact hyperfine interaction between the Rb valence electron and the ^129^Xe nucleus, and
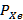
 the ^129^Xe polarization.


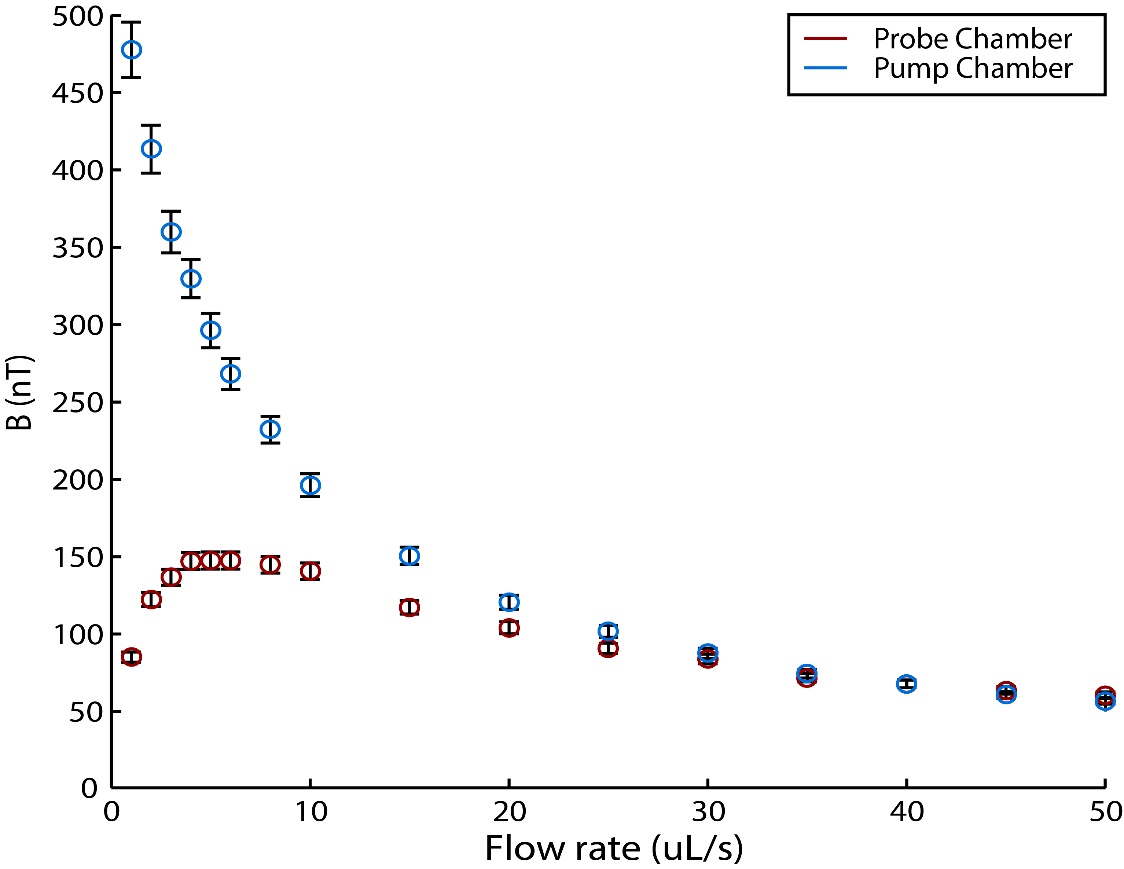


Supplementary Figure 3. Hyperpolarized ^129^Xe field as a function of total gas flow rate. Data in blue represent signals recorded in the pump chamber, while those in red were recorded in the probe chamber. The gas composition consisted of 800 Torr of Xe at natural isotopic abundance (26.4% ^129^Xe) and 800 Torr of N_2_. An optical pumping power of 70 mW was employed in the pump chamber in all measurements. All experiments were conducted with a pump chamber temperature of approximately 140 °C and a probe chamber temperature of approximately 110 °C.

**
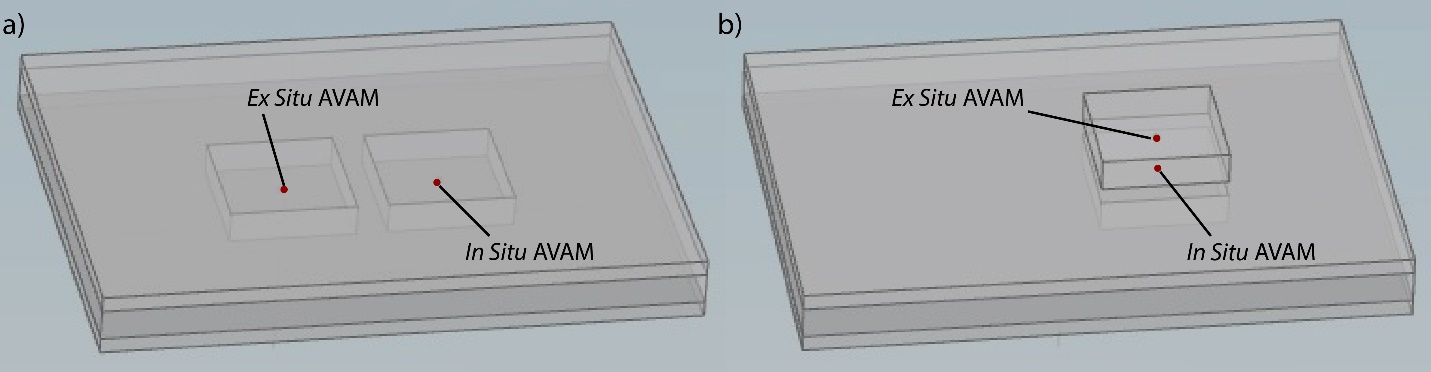
**

Supplementary Figure 4. Geometries employed in simulations of *in situ* magnetometer enhancement. a) Geometry employed in simulations of experiments shown in main text Figure 3. The *ex situ* magnetometer is embedded in the silicon microdevice a distance of 1 mm from the *in situ* magnetometer. b) Geometry affording a larger signal in the *ex situ* magnetometer. In this case, the *ex situ* magnetometer is a separate silicon and glass cell and is placed on top of the *in situ* magnetometer chamber. In both cases red dots indicate the point at which ^129^Xe magnetic fields are measured and are located at the geometric centers of the respective magnetometer chambers.

**Supplementary Note 1. Magnetometer Sensitivity Simulations**

The magnetic field detected by an *in situ* and *ex situ* magnetometer were simulated using the Magnetic Fields module of the COMSOL Multiphysics 5.0 software (COMSOL Group, Stockholm, Sweden). The simulation was conducted on a simplified geometry consisting of a pair of 4 mm x 4mm x 1 mm magnetometer cells separated by 1 mm etched through a 1 mm thick slab of silicon. The silicon was surrounded by 500 μm thick slabs of Pyrex on top and bottom. The simulated geometry is shown in Supplementary Figure 4a. The cell designated as the *in situ* magnetometer was designated as having a uniform arbitrary magnetization directed along the y-axis to represent the presence of hyperpolarized ^129^Xe. The magnetic fields at the geometric centers of each of the magnetometer cells were obtained and the *in situ* number was multiplied by 500 to simulate the Fermi-contact enhancement.

This yielded a signal in the *in situ* magnetometer which was 4900 times larger than that in the *ex situ* chamber. This enhancement is a bit smaller than the experimentally determined enhancement of 5300±200. To explain this discrepancy, we conducted additional simulations to determine the extent to which misalignments in the magnetometer laser affected the magnetic field measured in each chamber. These simulations were conducted by varying the location at which the magnetic fields were measured in each magnetometer chamber. This process revealed that changing the laser position by as little as 100 μm yielded *in situ* enhancements ranging from a factor of 4600 to a factor of 5300. Thus, any discrepancy between our experimental measurement and simulations can likely be explained by small optical misalignments.

An additional computational study was conducted to determine the enhancement factor when using an *ex situ* magnetometer with a more favorable location. An additional magnetometer cell built from 1 mm thick silicon and 500 μm thick pieces of Pyrex was placed above the *in situ* cell and the magnetic fields were measured. This geometry is shown in Supplementary Figure 4b and yielded an *in situ* enhancement by a factor of 2400. Thus, very large signal enhancements are attainable even when compared to an *ex situ* magnetometer with a more favorable location.
